# EEG dynamics during long-term treatment of depression in young female patients

**DOI:** 10.1192/j.eurpsy.2025.1331

**Published:** 2025-08-26

**Authors:** A. F. Iznak, ?. V. Iznak, E. V. Damyanovich, A. F. Beresneva, I. V. Oleichik

**Affiliations:** 1 Laboratory of Neurophysiology; 2 Department of Endogenous Mental Disorders and Affective States, Mental Health Research Centre, Moscow, Russian Federation

## Abstract

**Introduction:**

The need to study and clarify the neurobiological basis of depression is due to the widespread prevalence and heavy socioeconomic burden of this disease. In order to prevent relapses, it is recommended to continue treatment for depression for a long time after the relief of the main depressive symptoms. However, delayed EEG changes have been almost completely unstudied.

**Objectives:**

The aim of the study was to analyze the dynamics of spectral-coherent EEG parameters during long-term treatment for endogenous depression in young female patients.

**Methods:**

The study included 20 female patients aged 16-25 years who underwent quantitative clinical (using the HDRS-17 and GAF scales) and neurophysiological (multichannel resting EEG recordings with subsequent analysis of absolute spectral power and EEG coherence) examination three times: upon admission to hospital before the start of the course of treatment (at visit 1), upon discharge from the hospital at the stage of remission establishing (at visit 2) and one year after discharge from the hospital on maintenance therapy (at visit 3). The clinical and EEG changes were statistically assessed using the Wilcoxon test.

**Results:**

After the course of stopping treatment (at visit 2), there was a significant (p<0.01) reduction in depressive symptoms. This was associated with an EEG slowdown in the form of a generalized increase in the spectral power of theta-delta activity (2-4 Hz and 4-8 Hz), which was significant (p<0.05) in the frontal-central leads, and a significant (p<0.05) decrease in the alpha2 (9-11 Hz) and alpha3 (11-13 Hz) components of the alpha rhythm in the occipital zones. Further improvement in the clinical condition (according to the HDRS-17 scale) and social functioning (according to the GAF scale) was noted a year later (at visit 3), and was associated with the same EEG pattern, including a significant (p<0.05) increase of theta2 sub-band (6-8 Hz) spectral power in the central-parietal-occipital leads.

**Conclusions:**

The observed EEG slowdown is considered to be a reflection of a complex restructuring of brain activity into a mode more adequate for these patients, ensuring the suppression of depressive symptoms and restoration of the social functioning of patients, rather than traditionally considered general decrease of the brain functional state.

**Disclosure of Interest:**

None Declared

